# Almost 50 Years of Monomeric Extracellular Ubiquitin (eUb)

**DOI:** 10.3390/ph17020185

**Published:** 2024-01-31

**Authors:** Ivette Mendoza-Salazar, Ana Fragozo, Aneth P. González-Martínez, Ismael Trejo-Martínez, Rodrigo Arreola, Lenin Pavón, Juan C. Almagro, Luis Vallejo-Castillo, Francisco A. Aguilar-Alonso, Sonia M. Pérez-Tapia

**Affiliations:** 1 Unidad de Desarrollo e Investigación en Bioterapéuticos (UDIBI), Escuela Nacional de Ciencias Biológicas, Instituto Politécnico Nacional, Prolongación de Carpio y Plan de Ayala S/N, Colonia Santo Tomás, Alcaldía Miguel Hidalgo, Mexico City 11340, Mexico; ivette.mendoza@udibi.com.mx (I.M.-S.); ana.fragozo@udibi.com.mx (A.F.); agonzalezm2222@alumno.ipn.mx (A.P.G.-M.); ismael.trejo@udibi.com.mx (I.T.-M.); juan.c.almagro@globalbioinc.com (J.C.A.); lavallejos@ipn.mx (L.V.-C.); 2 Laboratorio Nacional para Servicios Especializados de Investigación, Desarrollo e Innovación (I + D + i) para Farmoquímicos y Biotecnológicos, LANSEIDI-FarBiotec-CONACyT, Prolongación de Carpio y Plan de Ayala S/N, Colonia Santo Tomás, Alcaldía Miguel Hidalgo, Mexico City 11340, Mexico; 3 Departamento de Inmunología, Escuela Nacional de Ciencias Biológicas, Instituto Politécnico Nacional, Prolongación de Carpio y Plan de Ayala S/N, Colonia Santo Tomás, Alcaldía Miguel Hidalgo, Mexico City 11340, Mexico; 4 Subdirección de Investigaciones Clínicas, Instituto Nacional de Psiquiatría Ramón de la Fuente Muñiz, Calzada México-Xochimilco 101, Colonia San Lorenzo Huipulco, Tlalpan, Mexico City 14370, Mexico; rarreola@imp.edu.mx; 5 Laboratorio de Psicoinmunología, Dirección de Investigaciones en Neurociencias, Instituto Nacional de Psiquiatría Ramón de la Fuente Muñiz, Mexico City 14370, Mexico; lkuriaki@inprf.gob.mx; 6 GlobalBio, Inc., 320 Concord Ave, Cambridge, MA 02138, USA

**Keywords:** extracellular ubiquitin, monomeric ubiquitin, immunomodulatory drugs, sepsis, biotherapeutic proteins

## Abstract

Monomeric ubiquitin (Ub) is a 76-amino-acid highly conserved protein found in eukaryotes. The biological activity of Ub first described in the 1970s was extracellular, but it quickly gained relevance due to its intracellular role, i.e., post-translational modification of intracellular proteins (ubiquitination) that regulate numerous eukaryotic cellular processes. In the following years, the extracellular role of Ub was relegated to the background, until a correlation between higher survival rate and increased serum Ub concentrations in patients with sepsis and burns was observed. Although the mechanism of action (MoA) of extracellular ubiquitin (eUb) is not yet well understood, further studies have shown that it may ameliorate the inflammatory response in tissue injury and multiple sclerosis diseases. These observations, compounded with the high stability and low immunogenicity of eUb due to its high conservation in eukaryotes, have made this small protein a relevant candidate for biotherapeutic development. Here, we review the in vitro and in vivo effects of eUb on immunologic, cardiovascular, and nervous systems, and discuss the potential MoAs of eUb as an anti-inflammatory, antimicrobial, and cardio- and brain-protective agent.

## 1. Introduction

Ubiquitin (Ub) was discovered in 1975 in bovine thymus and subsequently found in multiple organisms and tissues [1]. Ub is a small 76-amino-acid protein with a molecular weight of 8.6 kDa and a surface area of 4800 Å^2^. It is highly conserved across species. Indeed, human and mouse Ub are identical and differ from yeast by only two amino acids (96% sequence identity) [2], indicating a well-conserved role in regulating important cellular processes across diverse and evolutionarily distant organisms [2,3,4,5]. Ub is encoded by four different genes in humans; two of them, *UBA52* and *RSP27A,* encode for a single Ub fused to the ribosomal L40 and S27A proteins, respectively, whereas the other two, *UBB* and *UBC*, produce three and nine head-to-tail tandem Ubs with a C-terminal cysteine (C) or valine (V), respectively [6,7,8]. After expression, the polyubiquitins, as well as the C-terminal C or V extensions, are processed by specific cellular deubiquitinases (DUBs) to generate Ub [8].

In addition to Ub, other small ubiquitin-like (UBL) proteins are found in eukaryotic cells, including NEDD8, UFM1, ISG15, ATG8, URM1, SAP1, SUMO, and FAT10 [9]. Some UBLs have also been described in prokaryotes, such as the *Mycobacterium tuberculosis* prokaryotic-ubiquitin-like-protein (Pup). Nevertheless, contrary to eukaryotic Ub, Pup is an intrinsically disordered protein, which requires a specific set of enzymes to be conjugated to proteins by “Pupylation” [10,11]. Another prokaryotic UBL is the *Bacteroides fragilis* Ub (Bfubb), a protein that shares 63% identity with human Ub [12]. One of the main differences between human Ub and Bfubb is the substitution of the two C-terminal glycines (G) by a C, a simple modification that gives this bacterial protein the possibility to inhibit the intracellular reversible post-translational modifications of Ub, a process known as ubiquitination [12].

Although studies of Ub have mainly been focused on its intracellular functions, there are several lines of evidence showing that extracellular (eUb) can also regulate different cellular processes. eUb is found at nanomolar concentrations in serum, cerebrospinal fluid (CSF), lung, alveolar lining fluid, and urine in normal individuals [13]. The increase in eUb levels in extracellular fluids has been observed in several human pathologies such as multiple sclerosis, Alzheimer’s disease, sepsis, and Parkinson’s disease, as well as in burn injury [13,14,15,16,17,18]. Although the function of eUb in such diseases has been suggested to lessen disease progression, its MoA has only partially been elucidated and it is currently under debate. Some authors [19,20,21,22] have proposed that the chemotactic properties of eUb are attributed to its ability to activate the CXC chemokine receptor 4 (CXCR4), whereas others [19,23,24] have suggested that eUb is internalized into cells and used for the ubiquitination of intracellular proteins. 

This review aims to discuss the potential MoA of eUb and the functions that make this small and highly conserved protein a potential candidate for developing treatments for heart and brain tissue repair, as an immunomodulator, and as an antimicrobial agent. We also discuss the properties of eUb reported during the reproduction of different species. Further, we review applications of monomeric Ub as scaffolds to improve the uptake of active peptides into the cell [25]. Approaches of Ub as a biomarker have been discussed elsewhere [26] and are beyond the scope of this review. 

## 2. Ub Structure at a Glimpse 

**Figure 1:**
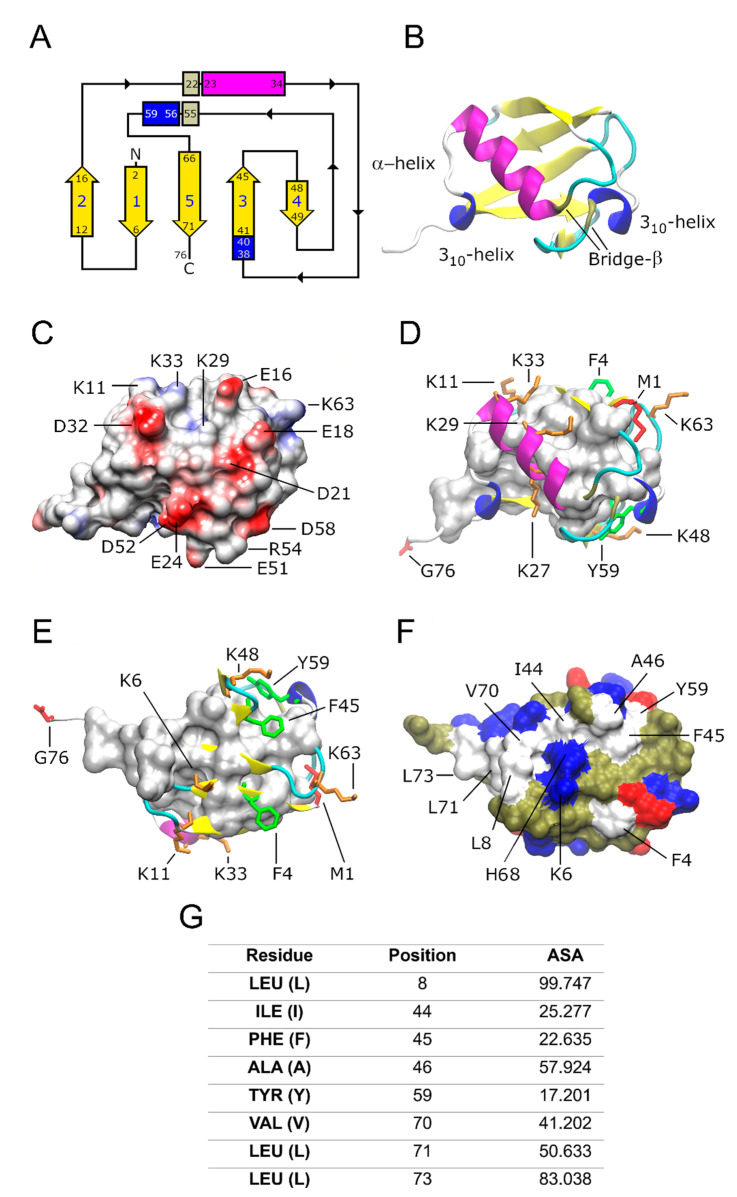
Ub topology and main structural features. (**A**) The ubiquitin fold topology shows an antiparallel five-stranded β-sheet (yellow color), two 3_10_ helices (blue rectangle), and one α-helix (magenta rectangle). Additional secondary structures are observed as an extended loop on the C-terminal with five residues (simple coils in a white loop) and six turn types I, I′, II, and IV (cyan color). The α-helix and the second 3_10_-helix are wrapped by a β-bridge forming a super secondary structure (gray rectangle), probably increasing the stability of the structure. (**B**) Ribbon representation of the frontal view of Ub. (**C**) The front view of ubiquitin shows the presence of multiple charged amino acids (but predominantly negative charges) suggesting that there is the possibility of a wide combination of protein–protein interactions: hydrogen bridge, cationic, dipole, induced dipole, and London dispersion forces. The Coulombic surface coloring was calculated with the UCSF CHIMERA program to generate the electrostatic potential according to Coulomb’s law [37]. (**D**) Frontal and (**E**) rear views of the fold show the functional side chains (spheres and tubes). M1 and G76 (red color), as well as K residues (orange color). Aromatic residues on the surfaces F4, F45, and Y59 (green color). (**F**) Solvent exposed surface of the rear view of Ub showing the hydrophobic core (white surface) formed by hydrophobic residues (A, V, I, L, F, and Y), acidic residues E and D are marked in red, and basic residues K, E, and H in blue. Polar, non-charged residues are represented in pale green. (**G**) % of accessible surface area (ASA) of the non-polar residues located on the hydrophobic patch of Ub. ASA values higher than 40 mean solvent-exposed residues. Panels A-F were edited in the Gimp program [38]. Panels B-F were built with information from references [39,40], using the Protein Data Bank (PDB) ID: 1UBQ and VMD program drawing methods [41].

The three-dimensional (3D) structure of Ub was first determined by Vijay Kumar et al. in 1987 using X-ray crystallography [27]. The characteristic fold of Ub consists of an antiparallel five-stranded β-sheet, two 3_10_ helices, and one α-helix (Figure 1A). The antiparallel β-sheet wraps the α-helix (Figure 1B). The complementary surfaces of the α-helix and β-sheet at the core of the protein are stabilized by hydrogen bonds and hydrophobic interactions [28,29].

The front view surface of Ub (Figure 1C) has a high content of negatively charged amino acid residues (E16, E18, D21, E24, D32, E51, D52, and D58) surrounded by positively charged residues (K11, K33, R54, and K63). These amino acid residues induce a negative electrostatic potential, suggesting that conformational shifts associated with charge recognition might be involved in the polyubiquitination process. 

Functional K residues (positions 6, 11, 27, 29, 33, and 48) form an “equatorial” band following the apparent direction from the C- to N-terminals of the α-helix (Figure 1D,E). Three of the seven functional K residues (positions 27, 29, and 33) are located on the α-helix (Figure 1D) and three K residues (6, 11, and 48) are located on the strands of the β-sheets on the rear view (Figure 1E). K63 is located on a loop close to the N-terminal (M1), away from the other K residues (Figure 1E). The described positive charged residues, side chains, and the N- and C-terminal residues, M1 and G76, play an important role in ubiquitination, as reviewed in [30], while the negatively charged ones do not have elucidated functions. There are multiple types of Ub conjugation via the lysine residues and N- and C-terminus of the protein, including monoubiquitination and polyubiquitination. Ubiquitination is regulated by sequential enzymatic activities of E1 (activating), E2 (conjugating), and E3 (ligating) ligases and it is counteracted by the activity of the deubiquitinase enzyme system [31]. 

In the rear view, residues from the antiparallel five-stranded β-sheet (I44, V70, and L71) and loops close to these β-sheets (L8, F45, A46, Y59, and L73) form a hydrophobic patch band (Figure 1F). By using Discovery Studio Visualizer [32], it was determined that most of these non-polar amino acids are solvent-exposed (Figure 1G). This exposed hydrophobic patch is known to bind non-covalently to other proteins [3,33]. These non-covalent interactions with Ub binding domains (UBDs) play critical roles in the regulation of various cellular processes, including protein degradation, DNA repair, cell cycle regulation, chromatin dynamics, stress response, gene silencing, membrane and protein trafficking, endocytosis, autophagy, and transcriptional and translational control [2,4,33,34,35,36].

## 3. Potential MoAs of eUb

The first suggested MoA for eUb was related to its ability to induce B-cell and T-cell activation, presumably by activation of β-adrenergic receptors (β-AR), given that these effects were blocked by propranolol [1]. Later, it was suggested that eUb interacts with the chemokine receptor CXCR4 [19,21,42]. After binding to CXCR4, it was seen that eUb promoted Ca^2+^ influx and decreased cAMP levels in human monocytes [19,42]. On the other hand, it was observed that activation of CXCR4 by eUb binding was abolished by Chicago sky blue 6B (CSB6B) dye, a small molecule that binds to the major interacting surface of Ub [43]. It was further shown that eUb binds to CXCR4 differently from stromal cell-derived factor 1 (SDF-1), also known as C-X-C motif chemokine 12 (CXCL12), the CXCR4 canonical ligand [44]. This differential binding to the receptor has functional consequences, as Ub inefficiently promotes the β-arrestin recruitment to CXCR4 [44].

However, the activation of CXCR4 by eUb was challenged by Job et al. [45] in 2015. The authors reported that eUb does not promote calcium influx or cAMP consumption in the same cell lines used by Saini and Tripathi in 2010 and 2014, respectively [19,42]. Moreover, in human macrophages, a discrete calcium influx was induced by Ub, but not being statistically different from the control (untreated cells) [46]. Furthermore, it has been shown that eUb decreases the proliferation of CaCo cells, which express low levels of CXCR4 [47], thus implying that eUb may have other MoAs besides CXCR4 interaction.

More recently, it high serum levels of Ub were found in mice exposed to X-ray, as well as in patients subjected to radiotherapy [22]. Jiao et al. [22]. described that, after radiation exposure, the expression of Ub is induced in the intestine and spleen tissues by activation of the interferon regulatory factor 1 (IRF1) transcription factor. Also, eUb treatment protected against X-ray lethality and reduced the deleterious effects of radiation on WT, but not in CXCR^null^ mice intestines. Although the results pointed out that the protective effects of eUb against X-ray exposure were via CXCR4, the authors did not exclude the possibility that this protection could be due to the interaction with other receptors [22]. 

EUb can be internalized into cells by diverse mechanisms [19,23,24,48]. For instance, it has been reported that eUb internalization is carried out by microtubule-based transport or a CXCR4-dependent mechanism, since pretreatment with nocodazole, a microtubule destabilizer, or AMD3100, a CXCR4 inhibitor, prevents Ub cell internalization [19]. Internalized Ub can promote apoptosis by inducing ubiquitination and degradation of STAT3, an important transcription factor linked to cell survival and proliferation [49], in a panel of leukemia and lymphoma cell lines [48]. In human monocytes, eUb is not only internalized but also used to ubiquitinate proteins, an effect that was more prominent after cell exposure to lipopolysaccharide (LPS) [23]. It is known that the total amount of ubiquitinated proteins decreases during sepsis [50] and that restoring ubiquitination by eUb internalization may protect cells from sepsis. In agreement with the latter suggestion, the accumulation of ubiquitinated proteins promotes macrophage-enhanced antimicrobial activity by inducing reactive oxygen species (ROS) production [51].

On the other hand, eUb uptake is also induced by stimulation of the β2-adrenergic receptor (β2-AR) with isoproterenol (ISO) in alveolar rat ventricular myocytes (ARVMs) [52]. Interestingly, β2-AR and toll-like receptor 4 (TLR4) interact with CXCR4 [53,54,55]. Since ISO and LPS can induce β2-AR and TLR4 internalization, respectively [56,57], ISO- and LPS-induced eUb-uptake is probably a consequence of eUb being binded to CXCR4 and then endocytosed as part of the TLR4/CXCR4 or β-AR/CXCR4 clusters. However, this hypothesis remains to be proven.

Another potential biological mechanism of eUb, described by Amoscato et al. [58], is its capability to suppress CD13-like peptidase activity towards a synthetic tyrosinase-derived peptide. These observations become relevant because CD13 has been suggested as a therapeutic target for inflammatory diseases such as multiple sclerosis [59] and inflammatory bowel diseases [60,61]. Interestingly, exopeptidase inhibition of CD13 leads to a sustained and slow cytosolic calcium increase as well as activation of the ERK, and Akt kinases [62,63], events that have been also observed due to eUb treatment [42].

However, whether inhibition of the proteolytic activity was by direct binding of Ub to CD13 remains to be elucidated. More recent data have shown the ability of Ub to bind to disordered peptides [64,65,66]. Indeed, degradation of the amyloid-β peptide (Aβ) by the insulin-degrading enzyme (IDE) is decreased when Aβ is bound to ubiquitin [64]. These observations raise the possibility that inhibition of the proteolytic activity of CD13 by Ub may be related to the ability of eUb to bind to the tyrosinase-derived peptide instead of CD13. Furthermore, the ability of eUb to bind to extracellular proteins brings a new scenario where eUb binds to extracellular peptides and/or proteins (such as cytokines) to activate/inactivate cellular responses, which comes as a new opportunity for research. Therefore, we hypothesize that Ub may bind to protein surfaces with (i) specific biochemical characteristics such as hydrophobic expositions and not-well-established hydrogen bonds, (ii) regions with water in not-favored interactions, or (iii) regions in dynamic mobility states (as unfolded or intrinsically disordered proteins) which are easily recognized by the eUb’s hydrophobic patch.

The fau ubiquitin-like (FUBI/MNSFβ) is a UBL molecule expressed as a fusion protein to the small ribosomal subunit protein S30 [67]. After cleavage by the USP36 [68], FUBI is secreted into the extracellular space [69]. FUBI, similar to ubiquitin, inhibits LPS-induced TNF-α production, suggesting an anti-inflammatory role of this protein. Interestingly, during the characterization of the interleukin 11 receptor (IL11-R) as a cell receptor for extracellular FUBI, the authors also found that ubiquitin binds to the same protein [70]. These observations suggest that IL11-R may also be regulated by eUb. This fact may have significant clinical repercussions since there is evidence that IL-11 is involved in the development of pathological conditions of the nervous system. The role of IL-11 in Alzheimer’s disease, autoimmune encephalomyelitis, multiple sclerosis, ischemic brain damage, and other diseases is studied in animal models [71]. In the case of relapsing–remitting multiple sclerosis (RRMS), IL-11 regulates inflammatory cell trafficking to the central nervous system (CNS) [72]. IL-11 is highly expressed in dendritic cells (DCs), neutrophils, and monocytes, and IL11-R in neutrophils, highlighting the role of IL-11 signaling in myeloid cells, which facilitates blood–brain barrier (BBB) disruption and the inflammatory cell migration to the CNS. RRMS patients have an increased expression of IL-11+ CD14+ monocytes, IL-11+ and IL-11R+ CD4+ cells, and IL-11R+ neutrophils in comparison to matched healthy control patients [72], so the blockade of the IL-11R receptor by eUb could be explored in preclinical research. During the writing of the present review, no information was found regarding the regulation IL11-R by eUb.

ISG15 is another UBL described to possess activities as an extracellular protein [73,74] (to better understand this UBL and its specific roles in biological systems, we recommend to the reader these three interesting reviews [75,76,77]). Specifically, extracellular ISG15 (eISG15) was described to induce IFN-γ production [74], and later the lymphocyte function-associated antigen 1 (LFA-1) was identified as an eISG15 receptor in immune cells [73]. ISG15 residues R99, T101, and T103 were identified as important amino acids for the binding of eISG15 to the LFA-1 subunit, CD11a [73]. Importantly, the authors found no effect on IFN-γ secretion due eUb treatment [73]. Although T103 in ISG15 structurally aligns with Ub T22, ISG15 R99 and T101 correspond to E19 and S20 in ubiquitin, respectively [73]. These observations strongly suggest that it may be a cooperative effect between R99, T101, and T103 of ISG15 to bind to LFA-1 and may explain why eUb fails to activate this receptor.

**Figure 2:**
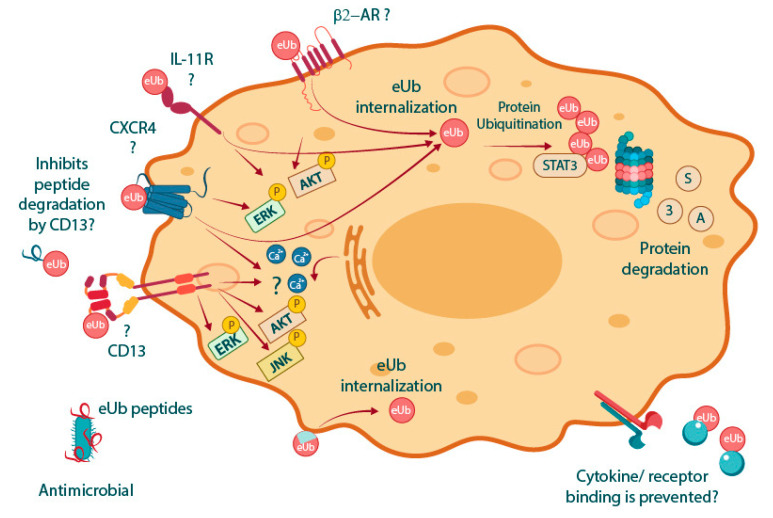
Several MoAs are proposed by the state of the art for eUb. The β-AR was suggested as the first cell receptor for eUb. Although the binding of eUb to the IL-11R has been reported, the biological relevance of this interaction has not been further explored. The interaction between Ub and CXCR4 is the most widely reported; however, there is still controversy regarding the effect of eUb signaling through this G-protein-coupled receptor (GPCR). Another extracellular receptor for eUb is CD13, whose proteolytic activity is inhibited by eUb. Interestingly, the four described potential receptors of eUb can promote ERK and Akt pathway activation, events that are known to be induced due to eUb treatment [42,43]. eUb is internalized, but it is unclear if this event is mediated by receptor-aided internalization or if eUb binds directly to membrane lipids owing to the hydrophobic patch. Once internalized, eUb can reprogram protein ubiquitination and degradation of STAT3 [48]. The ability of eUb to bind to several proteins and disordered peptides suggests that eUb may mediate biological responses by directly binding to extracellular structures, such as cytokines. On the other hand, some eUb peptides possess antimicrobial activity *vide infra*.

Figure 2 summarizes the MoAs of eUb. β-AR, CXCR4, IL11-R, and CD13 are potential extracellular receptors for eUb. Additionally, eUb internalization and reprogramming of the ubiquitination process have also been described. However, it is unclear whether eUb-uptake is dependent on a specific cell receptor. On the other hand, the presence of a hydrophobic patch surrounded by multiple positively charged amino acids suggests that eUb may interact directly with membrane lipids and perhaps be internalized passively, without interacting with any specific receptor.

Interestingly, the MoAs of eUb might include activities not only related to interaction with proteins. It is reported that eUb has intrinsic proteolytic activity against denatured β-galactosidase, but not the native protein, suggesting another mechanism of action of eUb in the removal of unfolded proteins [78,79]. Moreover, this protease activity has also been observed against collagen, gelatin, and fibrin, but not to other proteins such as human albumin [80,81]. These observations suggest that the Ub–protease activity may participate in the removal of unfolded proteins and damaged extracellular matrix in areas of inflammation to aid in the healing process. 

## 4. Biological Effects of eUb

### 4.1. Immune System

eUb levels are increased in the serum and plasma of patients with inflammatory diseases [82,83], suggesting a potential role of eUb during the inflammatory processes. Specifically, intravenous administration of eUb reduces TNF-α plasma levels, and the mortality induced by endotoxin in pigs [84]. In a lung polytrauma pig model, intravenous administration of Ub improves oxygenation and decreases circulating lactate levels as well as inflammatory cytokines (IL-8, IL-10, TNF-α, and CXCL12) in the pulmonary tissue [85]. Similarly, eUb enhances the Th2 cytokine response and improves pulmonary function in post-ischemic lungs when administered before reperfusion in rats [86]. On the contrary, in a model of ischemia-reperfusion heart injury, administration of eUb with an osmotic pump does not provoke any changes in circulating levels of IL-4, IL-10, or IL-13 [87]. Yet, it was able to protect against cardiac injury, which suggested that eUb’s anti-inflammatory properties may be restricted to sites of damaged tissue instead of having a systemic effect.

In human peripheral blood mononuclear cells (PBMCs), eUb prevents the LPS-induced TNF-α and IFN-γ production and promotes an increase in the anti-inflammatory cytokines IL-8 and IL-4 [16,88]. In contrast, in RAW264.7 cells, a murine macrophage cell line, eUb synergizes with LPS to induce TNF-α production [89], indicating that the anti-inflammatory properties of eUb may be cell-type dependent and/or species specific. On the other hand, eUb and a small Ub-derived peptide decreased the number of antigen-forming colonies of murine-spleen cells stimulated with sheep red blood cells (SRBC) [90,91]. In one of these works [91], the authors reported that the Ub50-59 peptide, with a rigid secondary structure, has a stronger immunosuppressive activity than the full-length protein, suggesting that Ub and its short-derived peptide may share a common receptor. Moreover, eUb can decrease the one-way mixed leukocyte in vitro reaction and improve skin graft survival, without any deleterious effect on body weight in mice [92]. Furthermore, an anti-inflammatory Th2 cytokine profile and an M2 polarization are provoked in human PBMCs, and macrophages incubated with eUb, respectively [88,93]. In macrophages, eUb has been shown to promote the anti-inflammatory M2 polarization, an effect that could be blocked by the addition of 100 µM of the CXCR4 inhibitor, AMD3100 [94]. Although this observation indicated that eUb-induced M2 polarization is via CXCR4, the high AMD3100 concentration used in that study makes it difficult to draw a solid conclusion since AMD3100 at doses higher than 10 µM has been shown to activate CXCR7 [95].

Although experimental data pointed out that eUb is a promising candidate to treat inflammatory diseases, the deleterious effects of this protein in cancer must be considered. eUb reduces apoptosis and promotes lung metastasis, as well as tumor progression of B16 melanoma in mice, which is related to increased matrix metalloproteinase-9 (MMP9) and vascular endothelial growth factor (VEGF) production [93,96]. Interestingly, an increase in anti-inflammatory cytokines was reported in this study. eUb does not affect the migration or apoptosis of the HepG2 cells [94], notwithstanding when HepG2 hepatoma cells reduced apoptosis and increased migration when co-incubated with eUb-pretreated macrophages [93,94]. Considering that eUb induces M2 macrophage polarization, it was suggested that eUb promoted tumor progression by inducing the tumor-associated anti-inflammatory process [94]. Therefore, in case of a possible use of Ub as an anti-inflammatory biotherapeutic, it should be important to consider the patient’s general health status before administration. 

Effects of eUb have been also reported on cell proliferation. Intraperitoneal administration of eUb in mice subjected to chemically-induced inhibition of hematopoiesis resulted in a quadruple increase in bone marrow cell count within 24 h. Notably, this effect contrasted with the reduction of peripheral blood cell count, suggesting that eUb could regulate stem cell activity, normalizing the release of functional cells into the bloodstream [97]. Moreover, in a recent investigation, it was described that eUb can modulate the erythroblast/megakaryocyte ratio and reduce cell size during bone marrow’s proliferative activity, accentuating Ub’s role in modulating hematopoiesis [97]. Studies involving the Ba/F3 cell line (a murine pro B cell line) demonstrated that Ub overexpression results in its secretion in these cells. The same study also established that the addition of eUb inhibits proliferation across multiple blood cell lines (HL-60, KT-3, U937, and Daudi) [48]. Collectively, these findings highlighted the potential of eUb as a key regulator in hematological cell proliferative processes. 

In the context of mouse tumor lung cells, eUb treatment failed to exert any significant impact on cellular proliferation. Neither ERK pathway activation nor STAT3 effects were observed, but there was a notable activation of AKT3 [98]. These observations suggest that Ub plays distinct roles across hematopoietic cells and tumor epithelial cells in proliferation. 

### 4.2. Nervous System

The neuronal cells are another potential target of Ub. In 1986 and 1987, Meyer et al. [99,100] showed that treatment with a specific anti-Ub antibody decreases sodium-dependent neurotransmitter transport in rat synaptosomes, an effect that is not related to cell polarization or antibody internalization. Although the authors suggested that there may exist a ubiquitination signal in the outer face of the cellular membrane necessary for neurotransmitter uptake, the possibility that free eUb may directly participate in such a mechanism should not be excluded. 

The role of eUb in brain repair has been also described. In swine, administration of eUb 30 min after brain injury (BI) reduces cerebral perfusion pressure, due to decreased intracranial but not arterial pressure, and reduces the intravenous fluid administration requirement during resuscitation after BI [101]. Interestingly, the authors [101], also found that eUb concentration in CSF increased after the treatment administration, suggesting that Ub can cross the BBB. Furthermore, it has been shown that eUb reduces contusion volume in rats [102] and promotes microglia/macrophage activation after BI [103]. Altogether, these observations suggested that after BI, eUb promotes the activation of phagocytic cells in the CNS to accelerate the brain healing process.

An interesting use for monomeric eUb has been proposed by Abarca-Castro et al. [104]. They suggested that monomeric Ub could be used as a therapeutic to limit the harmful effects on the neurodevelopment of offspring due to the inflammatory response caused in pregnant mothers by pre-eclampsia. They argue that pre-eclampsia, characterized by hypertension and organ damage during pregnancy, is linked to the offspring’s cognitive deficits, behavioral abnormalities, and neurodevelopmental issues [105], which significantly affect their development and adult life. The evidence indicated that the cholinergic anti-inflammatory pathway (CAP) could significantly impact the fetus’ and the newborn’s development by functioning as a neuroimmunology network facilitating internal monitoring. This pathway connects the CNS with the vagus nerve, regulating inflammation in the body [106].

In addition, Ub can disassemble amyloid-β42 aggregates in vitro and prevent uptake and cytotoxicity of the aggregates in SH-SY5Y cells when Ub is added to the cell culture [80]. Therefore, eUb may be useful to treat Alzheimer’s disease and pre-eclampsia, considering that in both pathologies amyloid-β42 aggregates are involved [107,108].

### 4.3. Cardiovascular System

Ischemic heart disease (IHD) is one of the primary causes of death worldwide. IHD is characterized by acute myocardial infarction due to acute lethal ischemia-reperfusion (I/R) injury, and cardiomyocyte death, which can result in heart failure [109,110]. In coronary heart disease (CHD), augmented levels of eUb showed a positive correlation with worsening CHD, suggesting that eUb could play a role, even as a biomarker, for CHD progression [111].

Due to the limited proliferative capacity of myocytes [112], it becomes relevant to prevent its apoptosis during heart damage. Previous studies have established that β-AR stimulation can induce apoptosis in cardiac myocytes through the activation of the glycogen synthase kinase-3 (GSK-3β) and mitochondrial pathways [113]. During in vitro and in vivo experiments, pre-treatment with eUb reduces β-AR-stimulated myocyte apoptosis by inhibiting activation of GSK-3β and the c-Jun N-terminal kinase (JNK), as well as suppressing cytosolic cytochrome c release, effects that are mediated by activation of the phosphoinositide 3-kinase (PI3K)/Akt pathway [52,114]. Additionally, eUb promotes the production of matrix metalloproteinase-2 and -9 (MMP-2 and -9) and the tissue inhibitors of MMPs (TIMPs) in cardiac cells exposed to ISO [87,114]. MMPs’ proteolytic activity participates in extracellular matrix remodeling, facilitating cell migration to promote tissue repair [115]. Although MMP2 overexpression is related to increased heart failure [116], the protective roles of this enzyme against cardiac hypertrophy [117] and against Angiotensin II-induced hypertension [118] have also been reported. Thus, induced MMP2 overexpression may be part of the protective mechanisms of eUb against heart failure.

In a mouse model of myocardial I/R injury, eUb treatment reduces apoptosis, oxidative stress, and mitochondrial fission, but increases mitochondrial biogenesis in a CXCR4-dependent manner [115]. The authors also reported that, in isolated hearts, eUb treatment reduces infarct size and restores heart function after I/R injury by preventing myocyte apoptosis [115]. Moreover, in another study, it was found that eUb reduces the area at infarct risk and improves heart function by increasing the percentage of fractional shortening and the ejection fraction of the heart after I/R. Additionally, eUb reduces the inflammatory response in the heart by reducing neutrophils and macrophage infiltration [87]. 

Cardiac angiogenesis, the process by which new blood vessels are generated, is also important for heart tissue repair after an IHD [119], and VEGF-A is a well-known regulator of this process [120]. It is reported that eUb promotes the expression of VEGF-A in cardiac microvascular endothelial (CMEC) cells via CXCR4 activation, proposing another cardiac-protective mechanism of eUb after IHD [121]. Furthermore, eUb promotes CMEC migration, a necessary process for cell repopulation in newly created blood vessels [121]. 

After heart tissue damage, cardiac fibroblasts (CFs) proliferate and produce extracellular matrix (EM) components to promote wound healing [122]. However, uncontrolled fibroblast proliferation and excessive EM production may result in a stiff scar which limits muscle contraction. CFs can differentiate into α-smooth muscle actin (α-SMA) or positive myofibroblasts (MFs) [123]; MFs are more contractile than CFs [122,124]. Thus, scar produced by this type of cells affects to a lesser extent heart contraction [123]. In 2018, it was shown that eUb, through its interaction with CXCR4, stimulates the activation of the ERK1/2 pathway in cardiac fibroblasts, which increases the production of VEGF-A and decreases expression of β3 integrin, influencing fibroblast-mediated activities such as angiogenesis [125]. Cell migration into wounds and fibroblast growth promoted by fetal bovine serum are likewise reduced by eUb therapy [125]. Furthermore, eUb increases the production of MFs [125], which in turn results in increased contraction of fibroblast-populated collagen gel pads [125]. In line with the previous study, it was discovered that eUb has no direct pro-proliferative effect on cardiac fibroblasts [126]. Instead, a truncated form of Ub (1-74), but not the full-length protein, inhibits the pro-proliferative effects of CXCL12 [126], a CXCR4 agonist known to enhance cardiac fibroblast proliferation [127]. Interestingly, Ub (1-74) is known to bind, but not activate, CXCR4 [21]. Ub (1-74) is the product of the processing of Ub (1-76) by the IDE [128]. Importantly, IDE inhibition blocks the conversion of Ub (1-76) to Ub (1-74) and restores SDF-1′s pro-proliferative effects in cardiac fibroblasts [126]. 

The evidence reviewed above indicates that eUb plays a crucial role in cardiac tissue repair and remodeling after ischemic heart disease. It stimulates the formation of new blood vessels, enhances cell migration and tube network formation, and regulates fibroblast behavior in heart tissue. Additionally, eUb has heart-protective effects and improves cardiac function following ischemia/reperfusion injury. Further research is needed to understand the underlying molecular pathways of eUb in the aforementioned processes. 

## 5. Antibiotic Effects of Ub

Indiscriminate use of antibiotics has promoted drug-resistant microorganisms, which nowadays have become a severe health problem worldwide [129]. Thus, finding new antimicrobial agents is an urgent need. Antimicrobial peptides (AMPs) are important components of the host defense against pathogens [130]. In 2003, Kieffer et al. described nicotine-stimulated chromaffin-secreted granules as a potential source of eUb in bovines [131]. Interestingly, the authors also found that Ub inhibits the growth of *M. luteus*, *B. megaterium*, and *N. crassa* with a minimal inhibitory concentration (MIC) of 60 µM [131]. Furthermore, synthetic peptides derived from the positively charged hydrophobic Ub c-term (Ub65-76) show higher growth-inhibitory capabilities than full Ub against bacteria, yeast, and fungi. In addition, Ub65-76 induced membrane destabilization in *A. fumigatus* and inhibits calcineurin phosphatase activity, a crucial enzyme in the regulation of hyphal growth and morphology in some filamentous fungi [131]. Like these observations, Alonso et al. [132] found that Ub incubated with lysosomal cathepsins but not full Ub or cathepsins alone induces a bactericidal effect against *Mycobacterium*, reinforcing the fact that Ub-derived peptides have higher antimicrobial activity than full-length Ub. In 2007, Jin-Young Kim et al. [133] obtained a small 4 kDa peptide from human amniotic fluid, identified as part of the N-term of Ub1-18 and named AFP-1. This peptide has antimicrobial activity at the µM range against a broad spectrum of bacteria, fungi, and yeast [133]. Interestingly, another N-term Ub peptide (Ub1-34) synergizes with the Ub65-76 synthetic peptide to suppress the growth of fungi and yeast [131]. Moreover, a truncated Ub form lacking the two c-term glycine residues (named by the authors cgUb) from the oyster *Crassotrea gigas* showed bacteriostatic activity against Gram-negative and -positive bacteria at the low µM range, but no hemolytic activity when exposed to human red blood cells [134]; Table 1 summarizes these findings. On the other hand, antimicrobial eUb-derived peptides are also produced by the V8 endoprotease of *Staphylococcus aureus* or by cathepsins secreted by activated leukocytes in the extracellular space [135]. Considering all the previous findings, it is clear that the full-length Ub has poor antimicrobial activity. However, Ub peptides produced in lysosomes and exosomes of macrophages and chromaffin cells [131,136] may be an important source of these AMPs.

## 6. Effects of eUb in Reproduction

It has been suggested that Ub is secreted to the extracellular space by the yeast *Pichia pastoris* as a response to cellular stress [137]. Although the role of eUb in yeast remains to be elucidated it has been shown that eUb reduces cell growth by promoting G2 arrest in *Schizoacharomyces pombe*, which can be abrogated by the addition of the proteasome inhibitor Lactacystin [138]. In this case, the authors hypothesized that eUb is internalized to cells, which results in the unprogrammed degradation of cell cycle proteins. A similar effect has been observed in the KT-33 human cell line, where eUb promoted STAT3 ubiquitination and degradation; these effects are also diminished by the addition of proteasome inhibitors [48]. These observations reinforce the reprogramming of the ubiquitin/proteasome pathway induced by eUb as another mechanism of action. 

In the marine invertebrate *Halocynthia roretzi*, extracellular ubiquitination of the 70-kDa main VC component (HrVC70) by a sperm extracellular ubiquitinating enzyme is relevant for egg fertilization (reviewed in [139]). This phenomenon can be promoted by the addition of Ub and ATP to the media and can be blocked by adding an anti-Ub antibody or proteasome inhibitors [140]. These observations indicate that degradation of HrVC70 by the extracellular ubiquitination/proteasome system is important during egg fertilization in this invertebrate. 

In boars, Petelak et al. (2019) [141] found an indirect correlation between the degree of ubiquitinated membrane proteins in the extracellular space of sperm and its capability to induce blastocyst formation in fertilized oocytes, effects that were improved by treatment of sperm with a Ub-blocking antibody [141]. On the other hand, embryo implantation can be reduced by administrating Ub-neutralizing antibodies in mice [142]. Interestingly, Ub has been found as a biomarker in the secretome during blastocyst formation and development in mice and in humans [143]. Together, these observations suggest a direct effect of the amount of eUb on sperm quality, related to its capability to promote blastocyst formation, as well as in blastocyst development in different mammalian species.

One of the first steps in angiosperm pollination requires the adhesion of pollen to the stigma. Then, the growth of pollen tubes through the pistil allows sperm cells to be discharged to the ovule. In 2006, Kim et al. [144], found that Ub was co-purified with the stigma/stylar cys-rich adhesin (SCA), from *Lilium longiflorum* pistils. The authors also reported Ub as an important protein to induce pollen adhesion to the stigma [144] and described that exogenously added Ub promotes the SCA-induced adhesion of pollen tubes. This suggests that eUb may have an important role during the early stages of pollination in *Lilium longiflorum*.

## 7. Biopharmaceutical Use of Ub

### 7.1. Development of Ub-Based Biotherapeutic

Biotechnological drug products are biological medicinal molecules obtained from live sources using genetic engineering. This group includes cytokines, growth factors, hormones, interferons, and regulatory peptides and proteins [145]. In addition to therapeutic proteins, specifically monoclonal antibodies, peptides are one of the biotherapeutics that have gained relevance in recent years for the development of biopharmaceutical products.

The difference between a peptide and a protein can be established based on their size, with proteins being those structures with 50 or more amino acid residues [146]. However, there are cases in which this division is not clear, such as insulin (5.7 kDa) or eUb (8.6 kDa), which can be considered as a long peptide or a small protein.

Recently, the number of naturally occurring peptides that are known to regulate physiological functions and that could serve as models for the development of biotherapeutics has increased [146]. Besides peptide-derived hormones (e.g., insulin, vasopressin, and gonadotropin-releasing hormone), there are neuropeptides (e.g., enkephalin, substance P, oxytocin) and antimicrobial peptides (e.g., human neutrophil peptides and human beta-defensins) [147,148]. Interestingly, several neuropeptides and antimicrobial peptides are produced by immune cells under inflammatory conditions or after antigenic stimulation and bind to GPCRs, which are expressed in different immune cells, including T cells, macrophages, monocytes, DCs, and neutrophils [149].

Peptides have the characteristics to overcome the main disadvantages of the two most relevant groups of current drugs, being more specific (and less toxic) than drugs with a small structure and having better bioavailability than therapeutic proteins (>100 kDa) [150]. Nevertheless, peptide-derived drugs must overcome some main challenges: (i) they should be as close as possible to endogenous human proteins to reduce the risk of immunogenicity; (ii) they should be parenterally administered to avoid extended proteolysis and acid degradation in the stomach, and (iii) their structural properties need to be improved because they have low half-lives and high conformational freedom [146,150].

The physicochemical and biological characteristics of eUb make it an attractive molecule for biotherapeutic development. First, Ub is a highly conserved protein in eukaryote cells [151], which reduces the probability of generating adverse events and/or therapeutic failure due to the induction of anti-drug antibodies (ADAs). Second, Ub is not glycosylated, thus avoiding low microheterogeneity owing to the reduction of glycosylated isoforms in the final product, which allows better quality control. Third, Ub is highly stable, with a T_m_ of 95 °C at pH 7.0 [152], which could be a desirable property for storing Ub at room temperature as a lyophilized powder and/or possibly in solution. 

The state of the art points out that Ub is an endogenous protein involved in regulating immune, nervous, and cardiovascular systems, which are related to illnesses with profound relevance; thus, it could represent an underestimated protein as a potential therapeutic agent. Therefore, we searched for ubiquitin-based biotherapeutics on DrugBank [153], Clinical Trials [154], and Cortellis Drug Discovery Intelligence [155]. No marketed biopharmaceutical products based on Ub were found in DrugBank at the moment of writing this review. On the other hand, 97 clinical studies using the search entry “Ubiquitin” were found on ClinicalTrials.gov. Twenty-three trials are focused on molecules to which MoA is indirectly related to Ub. Out of these 23 trials, 9 analyze the Ub levels (mRNA or protein concentration) as a biomarker, and 65 studies are related to proteins of the proteasome system except Ub, such as Ub carboxy-hydrolase L1 (UCH-L1), Ub-protein ligase E3A (UBE3A), and levels of ubiquitinated proteins in general, among others. Additionally, using the same searching criteria, 1293 entries were found in Clarivate Drug Discovery Intelligence with similar results: all the information found is related to biomarker development or to therapeutic usages of the proteins related to the ubiquitination system. 

Interestingly, Ub is not currently being developed as a biotherapeutic despite the preclinical evidence shown in this review. One possibility is that Ub could have pleiotropic effects that make it difficult to study its mechanism of action and its biological effects. On the other hand, cases of the indirect use of Ub as a biotherapeutic are described below.

### 7.2. eUb as a Component of Dialyzable Leukocyte Extracts (DLE)

DLEs are complex mixtures of low-molecular-weight peptides (<10 kDa) obtained from the lysis and dialysis of buffy coats from healthy donors [156]. DLEs have been used as co-adjuvants in the treatment of viral, parasitic, fungal, and mycobacterial infections, as well as primary immunodeficiencies, and allergies [157,158]. Transferon Oral^®^, a human DLE (hDLE) manufactured under good manufacturing compliance, regulates the production of the inflammatory cytokines TNF-α, IL-6 e, and IFN-γ and increases the percent of survival when orally administrated in a murine model of cutaneous herpes simplex virus-1 (HSV-1) infection [159]. In addition, this hDLE increases the percent of survival of puppies infected with canine parvovirus when subcutaneously administrated by decreasing circulating levels of cortisol and catecholamines and increasing plasma levels of norepinephrine and serotonin [160,161]. 

Since their discovery in 1950–1970, the mechanism of action of DLEs has been partially understood owing to their complex compositions. Vallejo-Castillo et al. [162] performed a peptidome analysis by mass spectrometry and found that Ub1-76 and Ub1-74 are two of the main components of Transferon Oral^®^. Additionally, Polonini et al. [163] identified the ubiquitin-40S ribosomal protein (also known as the 40S ribosomal protein S27a), and the ubiquitin-ribosomal protein L40 among the main components of Imuno TF^®^, a dialyzable extract obtained from pig spleen. Furthermore, Vallejo-Castillo et al. [162] performed a proof-of-concept murine HSV-1 infection assay and observed an increment of the percent of survival of HSV-1-infected mice orally administrated with eUb (0.750 µg/48 h during 10 post-infection days) with respect to the infected/not treated control. They hypothesize that orally administered Ub might stimulate the intragastric *vagus* nerve endings, favoring the activation of the anti-inflammatory vagal arch [162]. 

Although vast research is needed to clarify the in vitro and in vivo MoAs of Ub, the above information points out that Ub is an interesting biomolecule for the development of biotherapeutics. 

### 7.3. Use of Ubiquitin as Scaffolds

Ub has been suggested for use as a scaffold protein with the capacity to bind to different targets (Affilin^®^). Several binding proteins have been proposed as an alternative to the use of antibodies; Affilin^TM^ was initially described using γ-B-crystallin as a scaffold [164,165], and more recently, a ubiquitin dimer has been proposed as an ideal scaffold for binding multiple targets by introducing mutations in surface-exposed amino acid residues and creating a phage display library [45,166,167]. The use of ubiquitin as a scaffold lies in its physicochemical and biochemical properties as mentioned above. Because Ub does not have post-translational modifications, it may be easily obtained in a recombinant form in bacteria [167]; its poor half-life in circulation, however, might be one of its drawbacks—this could need linking to other proteins, like Fc or BSA [167]. To overcome these restrictions, Affilin^®^ has also been employed in combination with antibodies and fab in various forms as a bispecific molecule and pair to adenoviral vectors [168,169]. Multiple targets focused on cancer therapy have been evaluated; however, their in vivo evaluation is still necessary.

## 8. Concluding Remarks

The research on Ub has been mainly focused on its intracellular functions. However, many reports have shown that Ub has a biological activity as an extracellular protein. In this review, we have discussed the biological activities of eUb as an immunomodulator agent, cardio- and brain-protective agent, and antimicrobial protein; these effects are summarized in Table 2, Table 3 and Table 4. Although eUb has been postulated to act via activation of CXCR4, some authors have challenged this idea. In this regard, Ub possesses the capability to interact with diverse proteins, raising the possibility that eUb could act at diverse levels, perhaps via a variety of receptors/proteins beyond CXCR4. Furthermore, the “stickiness” of Ub may explain its ability to be internalized into cells as part of a cluster of Ub/membrane components. Once internalized, eUb may be able to promote the reprogramming of ubiquitination, which may affect a set of distinct cellular processes. The eUb effects seem to be pleiotropic, and it is restricted to some cell types. Moreover, anti-inflammatory (immunomodulatory) activities of eUb appear to be tissue and species-specific given that in some tissues or species this protein induces an anti-inflammatory program, and in others it works as a pro-inflammatory factor. 

Considering all the information reviewed here, it is interesting that a Ub-based biotherapeutic has not been developed to date, almost 50 years after its discovery. This could be in part because the study of Ub has focused mainly on its intracellular role or its extracellular pleiotropic functions, which, additionally, may be the causes of the existing controversy in its extracellular mechanism of action. As Ub research progresses and the functions of eUb are better understood, innovative biotherapeutics will likely be developed based on this interesting protein.

## Figures and Tables

**Table 1:** Antibiotic activities of ubiquitin and ubiquitin-derived peptides.

	MIC (μM) ^1^				
Pathogen	Ub1-76 [131]	Ub65-76 [131]	Ub65-76 + 10 µM Ub1-34 [131]	AFP-1 [133]	cgUbiquitin [134]
**Gram-positive** **bacteria**					
*Micrococcus luteus*	60	5	5	ND	ND
*Bacillus megaterium*	60	4	3	ND	ND
*Bacillus subtilis*	ND	NA	ND	ND	0.4
*Listeria monocytogenes*	ND	NA	ND	8	ND
*Staphylococcus aureus*	ND	NA	ND	8	4.7
**Gram-negative** **bacteria**					
*Escherichia coli*	ND	20	20	32	0.2
*Salmonella typhimurium*	ND	NA	ND	32	ND
*Pseudomonas aeruginosa*	ND	NA	ND	ND	0.6
*Shigella flexneri*	ND	ND	ND	ND	0.7
**Filamentous fungi**					
*Neurospora crassa*	60	10	4	32	ND
*Aspergillus fumigatus*	ND	30	15	64	ND
*Tricophyton mentagrophytes*	ND	20	7	ND	ND
*Trichoderma viride*	ND	10	7	ND	ND
*Botrytis cinerea*	ND	ND	ND	16	ND
*Fusarium oxysporum*	ND	4	ND	16	ND
**Yeast**					
*Candida albicans*	ND	15	7	8	9.4
*Candida tropicalis*	ND	15	7	ND	ND
*Candida glabrata*	ND	20	10	ND	ND
*Cryptococcus neoformans*	ND	15	7	8	ND

^1^ MIC is the minimal concentration inhibiting bacterial, filamentous fungi, and yeast growth. NA: no activity at 100 μM. ND: not determined.

**Table 2:** Summary of the in vitro biological effects of eUb.

Cell Line (Source)	Dose and Time of Treatment	Observed Effect	References
**Colon carcinoma cell line** **(CaCo; ATCC)**	0.02–2.0 µM 7 days	Decrease in cell proliferation.	[47]
**Human PBMNCs**	865 ng/mL 0.1–1 µg/mL	Decreases TNF-α production induced by LPS.	[16,88]
**Mouse macrophages (RAW 263)** **(RCB cell bank)**	0.1–10 µM 24 h	Synergizes with LPS to induce TNF-α production (100 ng/mL).	[89]
**Mouse splenocytes**	1–100 µg/mL	Suppresses the humoral immune response to SRBC. Inhibits mixed leukocyte reaction.	[90,91]
**THP-1 macrophages** **(Shangai Biology Institute)**	10 µg/mL 72 h	Induces M2 macrophage polarization with a decrease in secreted TGF-β and increased IL10.	[93]
**Murine melanoma B16** **(Shangai Research Center for Southern Model Organisms)**	200–800 ng/mL 24–96 h	Decreases apoptosis and promotes invasion by inducing MMP9 and VEGF production.	[96]
**Blood cells:** **-Myeloid cells (HL-60 and U937)** **-B cells (Daudi)** **-T cells (KT3, MT4, YTC-3 and MOLT4)** **(Nakarai Tesque, Kyoto)**	100 µg/mL 48 h	Decreases cell viability and induces apoptosis.	[48]
**Neuroblastoma cells (SH-Sy5y)** **(not stated)**	1.0–5.0 μg	Prevents amyloid-β1-42 and prevent aggregate cytotoxicity.	[80]
**Cardiac microvascular endothelial cells (CMEC)** **(not stated)**	20 μg/ml	Promotes VEGF-A expression.	[121]
**Mouse tumor cell lines (B16-F10 and 4T1)** **(ATCC)**	10 μg/ml	Promotes cell migration.	[98]
**Alveolar ventricular rat myocytes (AVRM).** **(freshly obtained from hearts of adult male Sprague-Dawley rats)**	10 μg/mL 30 min prior ISO	Protects AVRM from ISO-induced apoptosis via blockade of GSK-3β and JNK activation. Protects against hypoxia/reoxygenation-induced apoptosis in ARVMs.	[52,114,115]
**Adult rat cardiac fibroblast** **(freshly obtained)**	10 μg/mL	Reduced fibroblast migration and proliferation. Promotes differentiation to myofibroblasts.	[125]
**Cardiac fibroblasts** **(isolated from SHT and WKY rats)**	1–10 µM	Decreases SDF-1-induced cardiac fibroblast proliferation.	[126]
**H9C2 rat cardiac myoblast** **(cell line bank of the Chinese Academy of Sciences, China)**	0.1–1000 µg/mL 15 min prior 2 h hypoxia	Prevents apoptosis.	[170]
***Saccharomyces pombe***	25–100 μg/mL Early log phase addition	Reduced cell growth.	[138]

**Table 3:** Summary of the in vivo biological effects of eUb.

Animal (Source)	Dose and Time of Treatment	Observed Effect	References
**Skin graft in mice**	3.125, 12.5 or 25 μg of ubiquitin/h 14 days	Decreases the leukocyte reaction of the host vs. graft response. Decreases skin graft rejection.	[92]
**ISO-induced cardiomyopathy in mice**	1 μg/g 1 h before ISO infusion	Prevents myocyte apoptosis (decreases cytosolic release of cytochrome C).	[114]
**Mouse head trauma**	1.5 mg/kg	Promotes the recruitment of activated macrophages around areas of brain injury and improves recovery markers after mechanical damage to the brain.	[101]
**Irradiated mice**	100 µg/mL at 72 h after irradiation	Regulation of stem cell activity.	[171]
**HSV-1 infection in mice**	12.5 ng, 0.125 μg, 0.25 μg, 0.5 μg, 0.75 μg, 1.0 μg, or 1.50 μg of eUb in mixture.	Regulation of the production of proinflammatory cytokines TNF-α, IL-6, and IFN-γ.	[159]
***Lilium longiflorum***	Bovine eUb 150 and 200 ng per matrix	Promotion of pollen adhesion to the stigma (pollen tube adhesion enhancer).	[144]

**Table 4:** Summary of the ex vivo biological effects of eUb.

Source	Dose and Time of Treatment	Observed Effect	References
**Isolated hearts**	10 μg/L5 min post-ischemia 1 μg/g/h	Reduced apoptosis, oxidative stress, and mitochondrial fission, but increased mitochondrial biogenesis in a CXCR4-dependent manner. Reduced the inflammatory response in the heart by reducing the infiltration of neutrophils and macrophages.	[87,115]

## Data Availability

All data used for this work is included in the manuscript.
